# Moral foundations messaging to improve vaccine attitudes: An online randomized experiment from Argentina

**DOI:** 10.1371/journal.pgph.0003276

**Published:** 2024-11-15

**Authors:** Maike Winters, Sarah Christie, Hannah Melchinger, Nahuel Arias, Luciana Lirman, Angus Thomson, Saad B. Omer

**Affiliations:** 1 Yale Institute for Global Health, Yale University, New Haven, Connecticut, United States of America; 2 Yale School of Medicine, Yale University, New Haven, Connecticut, United States of America; 3 Peter O’Donnell Jr. School of Public Health, UT Southwestern Medical Center, Dallas, Texas, United States of America; 4 UNICEF Argentina, Buenos Aires, Argentina; 5 Irimi Company, Lyon, France; University of Michigan, UNITED STATES OF AMERICA

## Abstract

The uptake of routine childhood vaccinations has declined globally since the start of the COVID-19 pandemic, due in part to increased vaccine hesitancy among parents. The Moral Foundations Theory proposes six foundations which can be targeted to increase vaccine uptake. In this study, we tested whether a post by UNICEF with a purity violation message could affect vaccine attitudes among parents in Argentina, where routine immunization coverage has been declining since 2014. Using an online randomized controlled survey experiment, we included 1,511 parents with a child under 12 years in Argentina. Outcomes were measured on the Vaccine Trust Indicator (VTI). We found that the purity violation message significantly affected vaccine attitudes: 80% of the intervention group scored high on the Vaccine Trust Indicator, compared to 73% in the control group (coefficient: 0.33, 95% Confidence Interval 0.20–0.47). Vaccine messaging framed as a purity violation is a promising way to improve vaccine attitudes among parents.

## Introduction

Each year, childhood vaccines prevent an estimated 4 million deaths globally [1]. Vaccinations given between 2021 and 2030 are projected to save more than 50 million lives [1, 2]. Despite their life-saving properties, global coverage of routine childhood immunization (RI) has declined substantially, particularly since the start of the COVID-19 pandemic [3, 4]. Research has shown that reduced uptake of routine immunization can be due to a myriad of factors, from accessibility issues at vaccination centers to increased vaccine hesitancy among parents [5, 6].

Vaccine hesitancy can be described on a spectrum, from full acceptance and uptake of all recommended vaccines to refusal and delay of some to outright refusal of all vaccines [7]. The global majority of parents still get their children vaccinated but alarming increases in delayed and refused vaccinations have been reported worldwide [8–10]. Reaching vaccine hesitant parents with messages that resonate is no easy task, as fact-based messaging often has very little to no effect [11].

To that end, recent work has looked at Moral Foundations Theory (MFT) to target vaccine hesitancy [12, 13]. When faced with moral dilemmas, people can often not justify their stance: MFT argues that this is because these foundations, or intuitions, are activated before strategic reasoning take place [14–16]. This theory proposes six moral foundations that are universal in the world, but can vary culturally, namely: care, fairness, loyalty, authority, liberty, and purity [17, 18]. Care and loyalty have been shown to have a positive association with vaccine attitudes and vaccination intentions [19, 20]. On the other hand, liberty and purity (also called sanctity) have been found to play a role in vaccine hesitancy [12]. The liberty foundation values individual freedom, which in the case of vaccination hesitancy may take shape in the form of anti-vaccine mandate stances and an emphasis on personal choice [21]. In contrast, the purity value in vaccine hesitancy is more related to a preference for natural immunity and avoiding pathogens. It also relates to bodily contamination, and worries that vaccine ingredients are ‘impure’ [12, 22–25]. A strong emotion connected to the purity moral foundation is disgust [26]. Parental purity values have been associated especially with Human Papillomavirus (HPV) vaccination uptake and acceptance, likely because of the link with HPV and sexual activity [23].

Targeting these moral foundations in vaccine messaging may therefore be beneficial. In the Netherlands, a retrospective review of governmental vaccination messages found that messages targeting liberty, authority and purity were associated with increased uptake of routine immunization [27]. Vaccine messaging framed along the care moral foundation had no significant effect on vaccine uptake in the Netherlands [27].

While global routine immunization coverage has substantially decreased since the start of the COVID-19 pandemic, routine immunization coverage in Argentina has decreased since 2014 [28]. For example, coverage for the third dose of diptheria-tetanus-pertussis vaccine (DTP3) decreased from 94% in 2014 to 83% in pre-pandemic 2019 and 76% in 2021 [29]. The first measles dose was received by 95% of children in 2014 and 81% in 2021 [29]. This decline remains largely unexplained; however, studies have suggested that vaccine hesitancy may play a significant role in Argentina’s reduced coverage. A survey in Buenos Aires reported that higher educated mothers in Argentina were found to be more likely to be vaccine hesitant, echoing a similar finding from Australia, where higher socio-economic status was associated with vaccine hesitancy [30, 31]. Mothers tend to be targeted on social media by accounts that spread vaccine disinformation, appealing to various aspects of motherhood [32]. An analysis of the most prominent Instagram accounts in this space found that one of the techniques that are deployed is to appeal to the ‘intuitive mother’, in which maternal intuition trumps medical science [32]. This could be an indication that purity values play a role, as a healthy, pure lifestyle is deemed important by higher income mothers [30, 33, 34].

One way to tap into the purity value is through appealing to disgust. Previous research found that a violation of the purity moral by eliciting disgust resulted in higher acceptance of vaccines [20]. In this study, we therefore tested whether a purity violation message could improve vaccine attitudes among parents in Argentina.

## Methods

### Ethics statement

All study procedures were approved by the Yale Institutional Review Board (approval number 2000031351) on June 21^st^, 2022. Formal consent was obtained at the beginning of the survey using an online consent form. Only those who provided consent were able to proceed to survey questions.

### Study methods

We conducted an online survey of parents with at least one child under the age of 12 years in Argentina, between July 1–12, 2022. Recruitment for the survey was done through market research company CloudResearch, using their existing online survey panels. Two screening questions were posed to ensure that participants had at least one child under the age of 12 years. Participants who completed the survey received a small monetary incentive of USD2.70. Participants were presented with information about the study followed by an online consent form. Only those who consented to the study could proceed to the survey questions. The survey was anonymous, data were kept confidential on a secured server. The survey was fielded nationwide but was oversampled in 15 provinces (out of 24 provinces in total) that had especially low routine immunization coverage: Catamarca, Chaco, Córdoba, Entre Ríos, Formosa, Jujuy, La Rioja, Misiones, Rio Negro, Salta, Santa Fe, Santiago del Estero, Tucumán, Buenos Aires and Ciudad Autónoma de Buenos Aires (CABA). The survey comprised questions on demographics, the Vaccine Hesitancy Scale (a scale that has been validated in parent populations [35]) and whether the parents had their children vaccinated according to the national immunization schedule.

**Fig 1:**
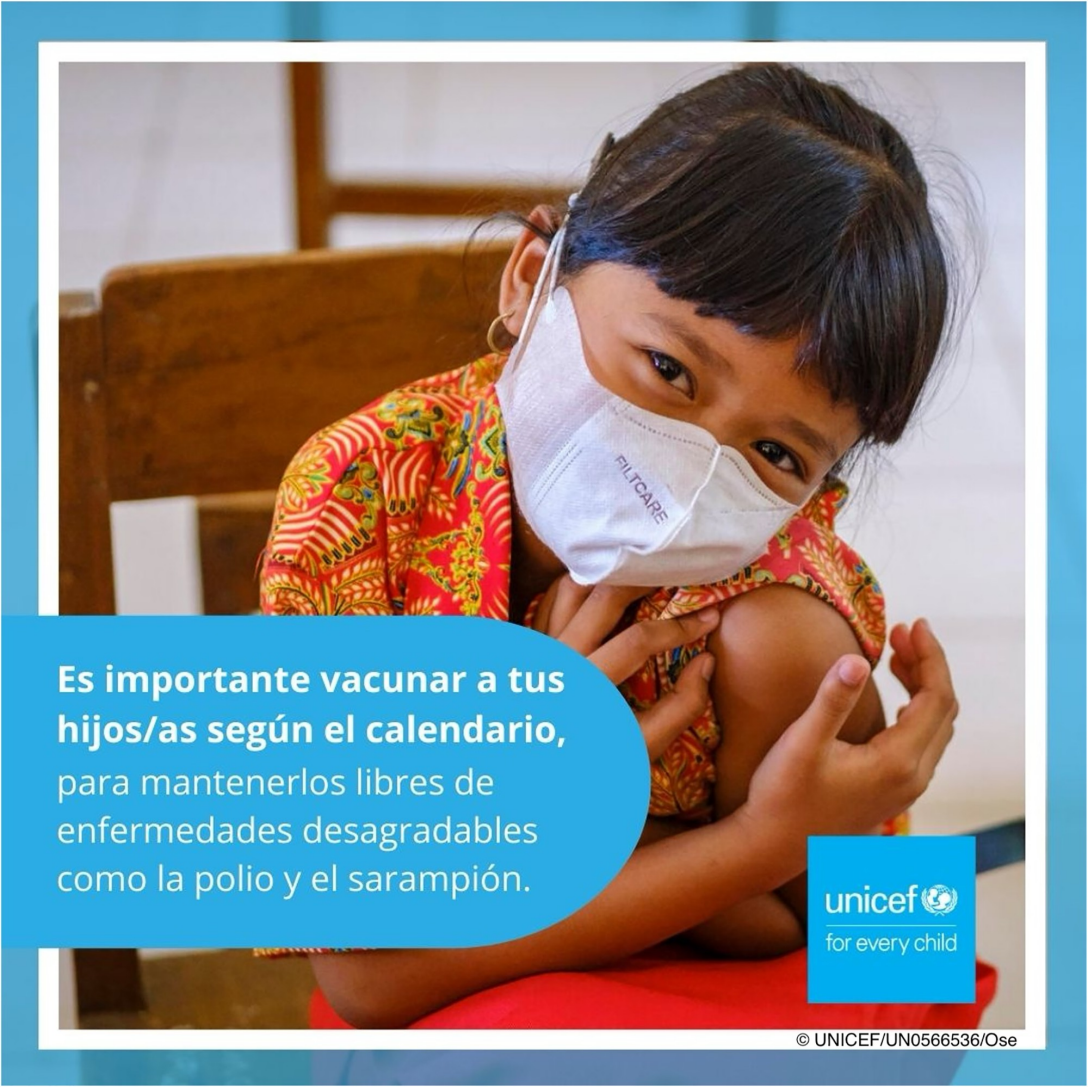
Online post shown to the intervention group. Republished from *UNICEF Immunization Roadmap to 2030* under a CC BY license, with permission from UNICEF, original copyright 2021.

Half of the participants (n = 756) were then 1:1 randomized to the intervention group, using a randomization block built into the online survey platform. The study was single blinded, as the participants were unaware of the randomization. While the control group continued with the final questions, the intervention group were asked to read the intervention post before answering the final questions. The intervention was a post from UNICEF, with a purity violation message. The message was created using the Moral Foundations Dictionary, a validated list of words that are associated with the moral foundations [36]. The Dictionary provides two lists per moral value; one of the virtue and one violation of the moral value. In case of purity, words like ‘disgusting’, ‘disease’, ‘indecent’ and ‘repulsive’ provide a violation of the moral value, whereas words like ‘pure’, ‘virgin’, and ‘pristine’ are virtuous purity words. As we aimed to test a violation of the purity value, we selected the words ‘disgusting’ and ‘disease’. The text of the post therefore read (in Spanish): ‘It is important to vaccinate your children following the schedule to keep them free from disgusting diseases like polio and measles’ (Fig 1). The message was written in Spanish and vetted by the UNICEF country office in Argentina, keeping in mind cultural sensitivities.

After reading the post, the intervention group answered the questions of the main outcome measure: the Vaccine Trust Indicator (VTI) [37]. As a non-vaccine-specific scale, the VTI has been developed to measure vaccine confidence among the general population. It has been validated in low- and middle income settings, where higher scores on the scale were associated with vaccine uptake [37]. This 6-item scale measures trust in various aspects of vaccines, each on a scale from 0–10: *‘Thinking about vaccination in general*, *would you say you are personally…’ (strongly against vaccination–strongly for vaccination)*, *‘I generally trust vaccine manufacturers or pharmaceutical companies (strongly disagree–strongly agree)*, *‘I generally trust the Ministry of Health’*, *‘I understand how vaccination helps my body fight infectious diseases’*, *‘I feel it’s important that I get vaccinated’ and ‘Vaccination forms part of a healthy lifestyle’*. The average score on the six items can be categorized into ‘low’ (<4), ‘medium’ (score between 4–7) and ‘high’ (>7) vaccine trust. The control group (n = 755) did not see any intervention post and instead went directly to the VTI questions noted above. The survey was originally written in English and was translated to Spanish by the UNICEF country office in Argentina. The translated survey was then checked by bilingual researchers of the study team.

#### Statistical analysis.

We summarized basic demographics descriptively and stratified by assigned group. We tested whether there was a difference in demographics between the control and intervention groups and thus whether the randomization was successful. The following demographic variables have been associated with vaccine trust and acceptance and were therefore analyzed [38, 39]: gender (male, female), age (18–24, 25–29, 30–34, 35–39, 40–44, 45+), education (primary, secondary, tertiary, postgraduate/professional), rural/urban living, employment status (employed, unemployed, domestic worker, student), religiosity (mean score on scale 0–10), political affiliation (left, neutral, right) and the mean score on the Vaccine Hesitancy Scale. Gender, education, urban living, employment, and political affiliation were analyzed using Chi^2^ tests, while religiosity and the Vaccine Hesitancy Scale were analyzed using a t-test. Next, we summarized the score on the Vaccine Trust Indicator (VTI) by categories (low, medium, high vaccine trust). We conducted linear regression analyses, using completed cases, to test whether there was a difference on the mean VTI score (ranged between 0–10) between the control and the intervention groups. We analyzed both the crude and the adjusted associations, the adjusted model included.

We fitted another linear regression model to test whether there was a different effect of the intervention between five provinces with the lowest routine immunization uptake, as measured by polio (IPV) and diptheria tetanus toxoid and pertussis (DTP3) vaccine uptake (Buenos Aires, Ciudad Autonoma de Buenos Aires, Catamarca, La Rioja and Santiago del Estero) and the rest of the country (based on UNICEF data) [28].

Lastly, we stratified the data in terms of children’s vaccination status (as reported by the parent) and analyzed the effect of the intervention among parents of undervaccinated and parents of vaccinated children. We used the question whether the respondents’ children had received routine childhood immunization according to the national schedule to categorize parents. Those who responded ‘yes’ were categorized as parents of vaccinated children. Parents who had immunized their children but not according to schedule, or whose children had received no vaccines, or some but not all vaccines, were categorized as undervaccinating parents. We used StataMP 17 for the data analysis.

## Results

**Fig 2:**
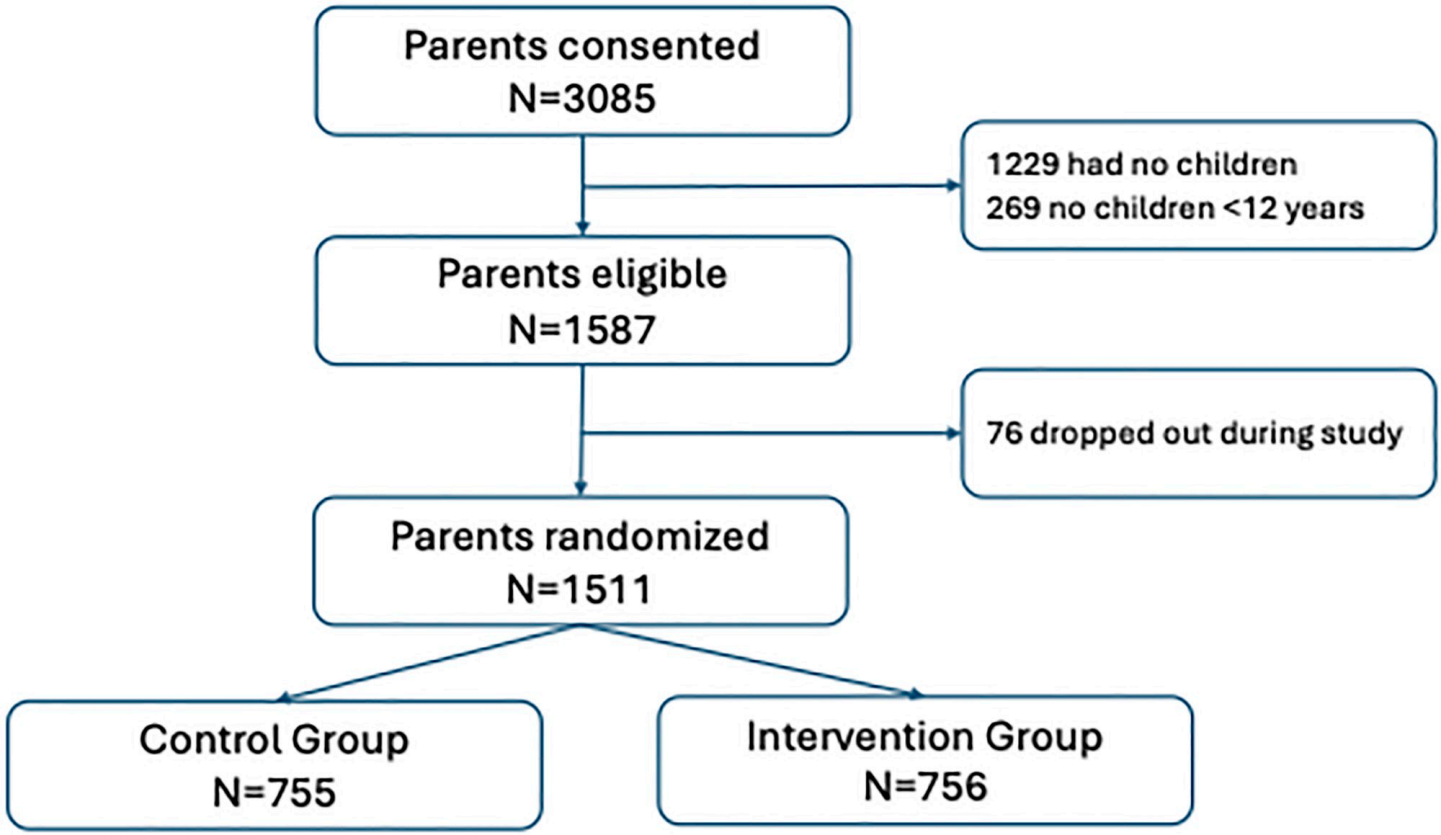
Flowchart of the study.

A total of 3,085 Argentinian parents consented to the study, of which 1,587 were eligible, see Fig 2. 1,511 parents with at least one child under the age of 12 years were randomized and completed the study, 76 parents (5%) started but dropped out during the study. In both the intervention and control group, there were slightly more males (56% in control group, 53% in intervention group), see Table 1.

**Table 1:** Demographics and Vaccine Hesitancy Scale by assigned group.

	Control (n = 755)	Intervention (n = 756)	P-value
**Gender**			
Male	422 (56%)	399 (53%)	0.233
Female	329 (44%)	352 (47%)	
**Age**			
18–24	129 (17%)	119 (16%)	0.321
25–29	148 (20%)	181 (24%)	
30–34	207 (27%)	184 (24%)	
35–39	150 (20%)	143 (19%)	
40–44	109 (14%)	119 (16%)	
45+	12 (2%)	10 (1%)	
**Education**			
Primary	14 (2%)	22 (3%)	0.513
Secondary	282 (37%)	267 (35%)	
Tertiary	413 (55%)	422 (56%)	
Postgraduate / professional	45 (6%)	44 (6%)	
**Urban/Rural**			
Urban	703 (93%)	695 (92%)	0.383
Rural	52 (7%)	61 (8%)	
**Employment**			
Employed	621 (82%)	613 (81%)	0.475
Unemployed	43 (6%)	58 (8%)	
Domestic	47 (6%)	43 (6%)	
Student	44 (6%)	42 (6%)	
**Political affiliation**			
Left	82 (11%)	78 (10%)	0.938
Neutral	484 (64%)	486 (64%)	
Right	189 (25%)	192 (25%)	
**Religiosity** (mean, SD)	2.87 (1.25)	2.89 (1.23)	0.785*
**Vaccine Hesitancy Scale** (mean, SD)	2.14 (0.55)	2.13 (0.60)	0.823*

*p-value from two-sided t-test

More than half the respondents had at least tertiary education (55% in the control group, 56% in the intervention group). More than 90% of the respondents indicated that they lived in urban areas, and more than 80% were employed at the time of the survey. In both the control and the intervention group, 64% said they were neutral in their political affiliation. There was no difference between the control and the intervention group in terms of religiosity or score on the Vaccine Hesitancy Scale. A total of 111 parents (7% of the sample) was categorized as undervaccinating their children, which included not vaccinating their children at all, as well as delayed and partial vaccination.

**Fig 3:**
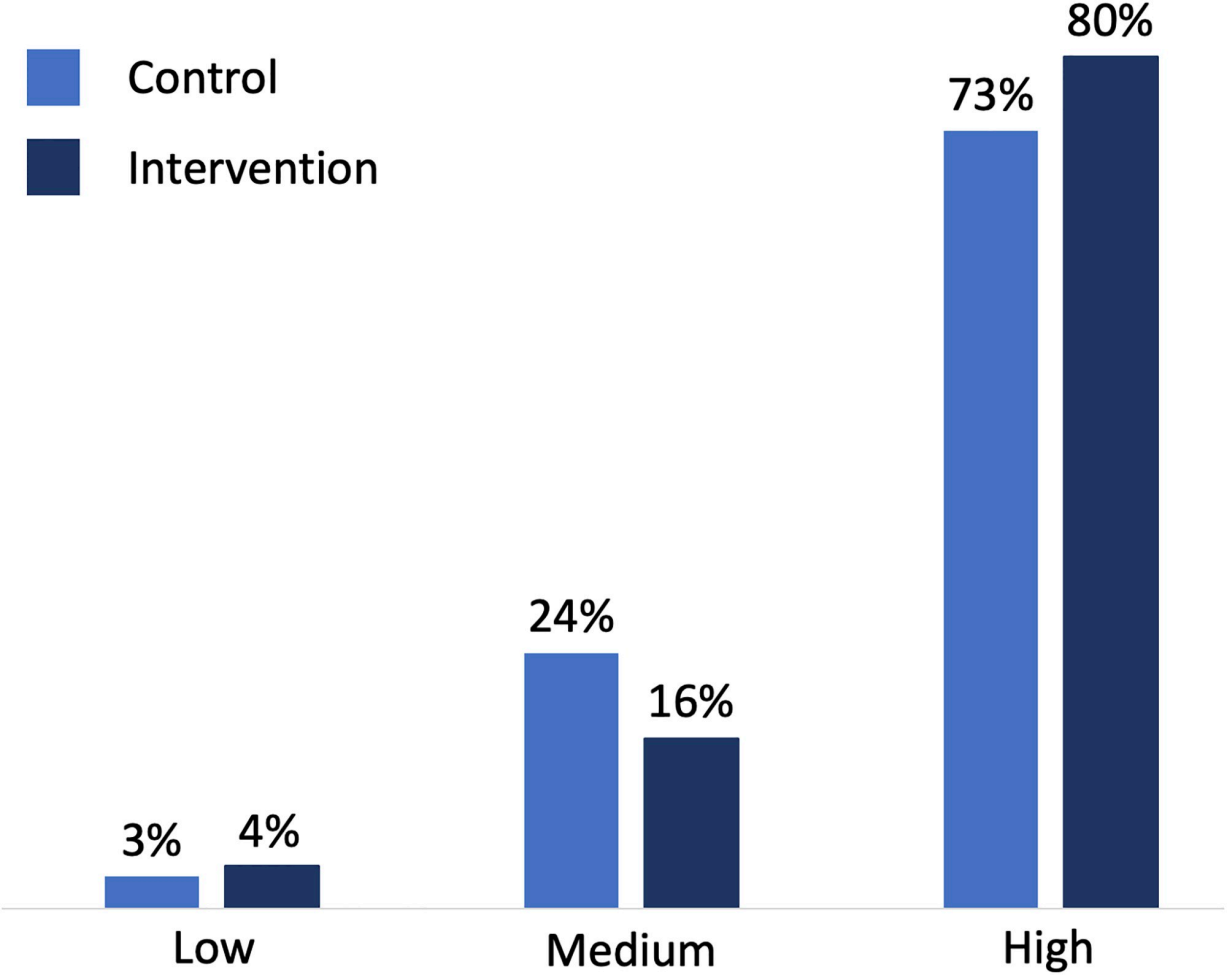
Score on the Vaccine Trust Indicator by assigned group.

Categorizing the Vaccine Trust Indicator score, the intervention group scored higher than the control group (80% intervention group scored high versus 73% in the control group) (Fig 3). The difference was mainly between the categories ‘medium’ and ‘high’–the control group had a larger share scoring medium than the intervention group (24% versus 16%).

The intervention post had a strong positive effect on the score of the Vaccine Trust Indicator; linear regression modeling shows an increase of 0.33 points on the VTI (95% Confidence Interval (CI): 0.20–0.47) after adjusting for various covariates, see Table 2. There was no difference in the effect of the intervention between the five lowest routine immunization coverage provinces and the rest of the country (adjusted coefficient -0.01, 95% CI -0.14–0.13).

**Table 2:** Effect of the intervention on the Vaccine Trust Indicator score.

	Coefficient (95% CI)	p-value	Adjusted* coefficient (95% CI)	p-value
**Group**				
Control	Reference		Reference	
Intervention	0.35 (0.16–0.54)	0.000	0.33 (0.20–0.47)	0.000
**Low RI coverage**				
Lowest	0.07 (-0.12–0.26)	0.467	-0.01 (-0.14–0.13)	0.913
Rest	Reference		Reference	

*adjusted for age, gender, education, rural/urban, employment, religiosity, political affiliation, vaccine hesitancy score

CI = Confidence Interval

When stratifying by children’s vaccination status, we find a similar strong positive effect of the intervention post among parents of vaccinated children (adjusted coefficient 0.32, 95% CI 0.18–0.46), see Table 3. Among the parents of undervaccinated children however, the sample size was likely too small to detect a significant effect (adjusted coefficient 0.42, 95% CI -0.07–0.90).

**Table 3:** Effect of the intervention stratified by children’s reported vaccination status.

	Parents of vaccinated children	P-value	Parents of undervaccinated children	P-value
	Adjusted Coefficient (95% CI)		Adjusted* Coefficient (95% CI)	
**Group**				
Control	Reference		Reference	
Intervention	0.32 (0.18–0.46)	0.000	0.42 (-0.07–0.90)	0.095

*adjusted for age, gender, education, rural/urban, employment, religiosity, political affiliation, vaccine hesitancy score

CI = Confidence Interval

## Discussion

Our study demonstrated the effectiveness of a morally framed message in improving vaccine attitudes. We found that a post with a purity violation message led to significantly higher trust in vaccines (0.33 points on the Vaccine Trust Indicator 10-point scale) among parents with at least one child under the age of 12 years in Argentina. This effect was especially seen among parents of vaccinated children, with no detectable effect among parents of undervaccinated children given the small sample size.

While most previous research thus far around MFT and vaccine hesitancy has been correlational, our randomized controlled experiment has shown the potential impact of targeting the purity moral foundation. This is in line with another experimental study, where a purity violation message in the form of a narrative of a Tinder date followed by a picture of genital warts, increased HPV vaccination intentions [20]. An observational study looking at MFT in 3 million English posts on Facebook public pages, found that while purity was the least used moral foundation in the posts, it was associated with an increase in likes [40]. On the other hand, posts framed along the care and fairness moral foundations were most used, but related to a decrease in likes [40]. In Utah, Mormon leaders appealed to spiritual purity by calling the COVID-19 vaccines a “literal godsend”. An uptick in vaccinations was registered shortly thereafter [41]. A retrospective analysis of vaccination brochures in the Netherlands found that messages around purity, such as ‘we believe it’s important that children get a wholesome start in life’, had a small positive effect on vaccination uptake [27].

Purity can be described in many different ways, for instance through a preference of natural immunity and avoidance of bodily and spiritual contamination [22, 23, 42]. It has also been described as a rejection of new things, such as new vaccines [42]. Furthermore, purity has been associated with conspiracy beliefs, indicating a tendency to reject science in general [43]. The broad scope of what could be regarded as appealing to a purity value, has also drawn criticism [44]. As our study focused on a purity violation in the form of disgust, it would be valuable to test moral messaging that target other aspects of purity.

While our study was not powered to detect an effect among parents of undervaccinated children, effects of moral framing might differ depending on the moral foundations and levels of vaccine hesitancy in different populations [42]. A study creating hesitancy profiles based on an analysis of more than 6 million tweets about COVID-19 and HPV vaccines, found that purity was associated with skepticism towards HPV vaccination [45]. For COVID-19 vaccines, it was mostly the profile of those who spread misinformation (i.e., the ‘misinformers’) that had a positive association with purity [45]. The misinformers were generally found to have stronger moral stances than other profiles, which could imply that moral messaging may resonate more strongly as well with such individuals. Future research could test various moral messaging strategies on different hesitancy profiles.

Taken together, pro-vaccination messages with a focus on purity violations could be used to enhance vaccine trust, attitudes and intentions [20]. Developing these messages should be done with great care, be informed by behavioral insights and targeted towards the key audience of the messages. For instance, the graphical picture of genital warts in the O’Marr et al. study [20] was successful in increasing vaccination intentions among young people. The same results may not be achieved (or even backfire) in other target populations. Our message on ‘disgusting diseases’ should also be used with great care, as unintentional stigmatization of people who have contracted measles or polio should be avoided.

### Strengths and limitations

A major strength of this study was the ability to target parents and to focus on parents living in provinces that have lower routine immunization coverage in Argentina. Using a randomized controlled survey experiment is a strong study design to detect effects of an intervention. Our study also demonstrates the usefulness of the Vaccine Trust Indicator as a short scale to measure general attitudes towards and trust in various aspects of vaccines and vaccination stakeholders [37].

However, there are various limitations associated with the study. First, while most Argentinian parents are online, our survey likely missed those who have no internet connection. Our sample was also higher educated compared to the Argentinian adult population. The oversampling of the low vaccine coverage provinces, may have limited the representativeness of our sample. While we were able to randomize, there may have been unmeasured confounding. The intervention post comprised not only the purity violation text, but also a picture of a happy child who just got vaccinated, as well as the UNICEF logo. This may also have been responsible for the observed effect of the intervention. In our study the control group did not see any messages. Their experience of the study could therefore have been different compared to the intervention group, which could have potentially biased the results. Future studies could test the same image with different phrasings of a purity violation to determine what the driver of change is, or a control message with the same imagery. The study was conducted in partnership with UNICEF, a potentially credible voice in the childhood immunization space, which also could have influenced the results. Lastly, we used a crude method of identifying parents of undervaccinated children by grouping together parents whose children have not received any vaccines with those whose children are partially vaccinated or not following the national schedule. Vaccine decision making is complex, and future studies should ensure statistical power to be able to detect differences between various vaccination levels.

Despite these limitations, the findings indicate a strong effect of purity messaging on vaccine trust among parents in Argentina. This has promising implications for future message framing for health agencies charged with strengthening childhood vaccination coverage.
